# Reduced abundance and earlier collection of bumble bee workers under intensive cultivation of a mass‐flowering prairie crop

**DOI:** 10.1002/ece3.2856

**Published:** 2017-03-12

**Authors:** Paul Galpern, Sarah A. Johnson, Jennifer L. Retzlaff, Danielle Chang, John Swann

**Affiliations:** ^1^ Faculty of Environmental DesignUniversity of CalgaryCalgaryABCanada; ^2^ Department of Biological SciencesUniversity of CalgaryCalgaryABCanada

**Keywords:** agricultural intensification, *Bombus*, bumble bee, Canadian Prairies, canola, mass‐flowering crop, oilseed rape, phenology, pollinator conservation

## Abstract

One of the most commonly seeded crops in Canada is canola, a cultivar of oilseed rape (*Brassica napus*). As a mass‐flowering crop grown intensively throughout the Canadian Prairies, canola has the potential to influence pollinator success across tens of thousands of square kilometers of cropland. Bumble bees (*Bombus* sp.) are efficient pollinators of many types of native and crop plants. We measured the influence of this mass‐flowering crop on the abundance and phenology of bumble bees, and on another species of social bee (a sweat bee; *Halictus rubicundus*), by continuously deploying traps at different levels of canola cultivation intensity, spanning the start and end of canola bloom. Queen bumble bees were more abundant in areas with more canola cover, indicating that this crop is attractive to queens. However, bumble bee workers were significantly fewer in these locations later in the season, suggesting reduced colony success. The median collection dates of workers of three bumble bee species were earlier near canola fields, suggesting a dynamic response of colonies to the increased floral resources. Different species experienced this shift to different extents. The sweat bee was not affected by canola cultivation intensity. Our findings suggest that mass‐flowering crops such as canola are attractive to bumble bee queens and therefore may lead to higher rates of colony establishment, but also that colonies established near this crop may be less successful. We propose that the effect on bumble bees can be mitigated by spacing the crop more evenly with respect to alternate floral resources.

## 1 Introduction

Wild bees are declining, putting at risk the pollination services they provide to plants (Cameron et al., 2011; Kerr et al., 2015). One of several identified threats to bees is the intensification of cropping on agricultural land (Rundlöf, Persson, Smith, & Bommarco, 2014). In many agroecosystems, changes in agricultural practices have led to the reduction of uncultivated areas that provide a more diverse and continuous array of flowers for pollinators throughout the season (Leong, Ponisio, Kremen, Thorp, & Roderick, 2016). Mass‐flowering crops (MFCs) may buffer any landscape change impacts on pollinators through the substantial pulse of floral resources that they provide (Stanley & Stout, 2013; Westphal, Steffan‐Dewenter, & Tscharntke, 2003). However, the effects on pollination services of agricultural intensification, such as replacing uncultivated and seminatural areas with mass‐flowering monocultures, and the impacts of this less diverse and temporally discontinuous forage, require further investigation.

Bumble bees (*Bombus* sp.) are efficient pollinators of many types of flowers and may be affected by MFCs in a variety of ways. The importance of floral availability for bumble bee colony and reproductive success has been extensively reported (Bowers, 1986; Pelletier & McNeil, 2003; Schmid‐Hempel & Durrer, 1991), but under which conditions increased floral availability will be a net benefit to pollinators and pollination services remains an important question. The increased nectar and pollen from MFCs can improve colony growth rates, which may lead to a dynamic response in queens via increased production of reproductive castes earlier in the season (Bowers, 1986; Pelletier & McNeil, 2003; Rundlöf et al., 2014). However, there is evidence that MFCs may fail to provide a reproductive advantage when alternate food resources are not available after the mass flowering period (Westphal, Steffan‐Dewenter, & Tscharntke, 2009). The expansion of fields and the cultivation of seminatural areas that eliminate alternate floral resources for bees may thus remove any potential beneficial effects of MFCs for pollinators (Garibaldi et al., 2011, 2014; Kennedy et al., 2013). In addition to the destruction of alternate food resources through cultivation of seminatural areas, the presence of MFCs can also have indirect effects on ecosystem function. Oilseed rape, an MFC, can dilute pollination services to wild plants (Holzschuh, Dormann, Tscharntke, & Steffan‐Dewenter, 2011), leading to a feedback loop of reduced reproductive success of both plants and bees (Persson & Smith, 2013).

Mismatches between flowering times and the presence of pollinators have contributed to losses of species diversity in bee communities (Bartomeus et al., 2013; Burkle, Marlin, & Knight, 2013). To address this, planting flowers that are complementary with the flight seasons of focal bee species has been recommended as a conservation measure (Russo, Park, Gibbs, & Danforth, 2015). For MFCs, it is likely that any benefit of the floral pulse for bees will depend on the timing of bloom. For bees with shorter flight seasons, MFCs may bloom outside a window of opportunity to exploit the resource. For social bees, such as bumble bees, mismatches between the timing of flight season and forage availability could lead to a scenario where the resource pulse creates a sink habitat. By providing abundant resources to encourage colony establishment and growth, but giving way to a landscape devoid of alternate floral resources when reproductive castes are being reared, MFCs may ultimately lead to failed colonies. The potential for impact of phenological mismatches of this sort suggests that both the timing of bee flight seasons and crop bloom (collectively referred to, here, as phenology) should be examined when evaluating the conservation implications of MFCs.

**Figure 1:**
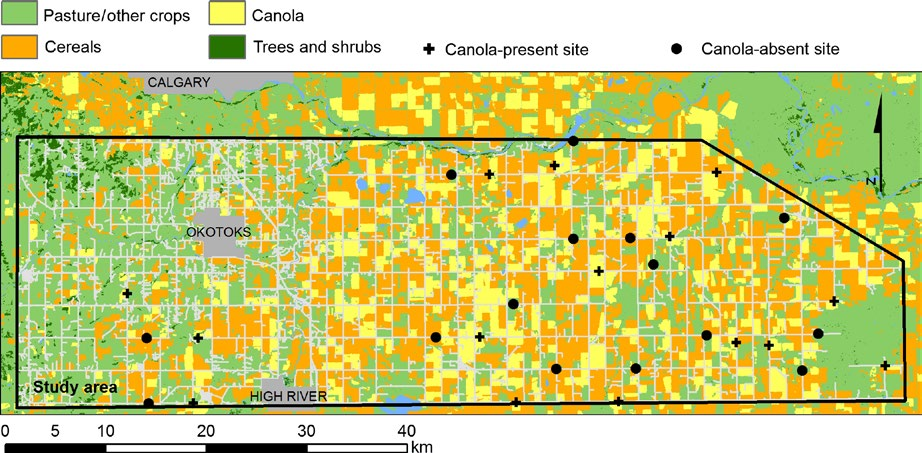
Study area near Calgary, Alberta, Canada, indicating locations of 15 sites adjacent to canola (canola‐present; 20%–95% canola cover within 250 m) and 15 sites not adjacent to canola (canola‐absent; <2% canola cover within 250 m). Field spatial data from Canada annual crop inventory (Agriculture and Agri‐Food Canada, 2015). Gray linear features indicate the extensive road network

Canola is a cultivar of oilseed rape (*Brassica napus*) and is an MFC widely grown throughout the Canadian Prairies and the North‐Central United States. Agricultural activities throughout this area are typically highly intensive, with much of the available land surface under the annual cultivation of either canola or cereal crops (Agriculture and Agri‐Food Canada, 2015). In contrast to many parts of Europe, where studies of wild bees and MFCs have mainly occurred (Warzecha, Diekötter, Wolters, & Jauker, 2016; Westphal et al., 2003), prairie agroecosystems tend to be dominated by large rectangular fields, hedgerows are seldom present, and in many regions, there are few other trees, shrubs, or seminatural land covers (e.g., Figure 1).

In this study, we use canola fields in the Canadian Prairies to examine the effects of MFC intensity on bee phenology and abundance by measuring bee presence along a gradient of canola exposure. Canola flowers attract many species of bee, and there is evidence that both nectar and pollen offer nutritional benefits (e.g., Westcott & Nelson, 2001). We focus on canola cropping intensity because it is a variable that farmers have the ability to manipulate at relatively low cost, facilitating conservation management solutions. We ask whether canola has an effect on the phenology and abundance of bees, by testing the hypothesis that bumble bee colonies will respond demographically to the increased food availability provided by canola. We predict that (1) bumble bee workers will have an earlier peak in their date of collection in landscapes with more canola, if food availability results in better provisioned colonies; and (2) after canola has finished blooming, bumble bee workers and new queens will be more abundant in landscapes where canola was planted, if there has been a positive effect of the canola food pulse. We also test the hypothesis that other bees with social life histories and a flight season spanning canola bloom will exhibit the same response. We predict that (3) the sweat bee *Halictus rubicindus,* which meets these criteria, will exhibit similar patterns in abundance and date of collection.

## 2 Materials and methods

### 2.1 Study design

Our study area is located in Southern Alberta (50.663°N, 113.483°W) in the Canadian Prairies and encloses a region (88 km by 25 km) where the cultivation of cereals and canola is spatially intensive (Figure 1). Trees and shrubs are sparsely distributed throughout this grassland region, and pasture and road margins represent the most common types of uncultivated land. Field sizes are large, as is typical in prairie agroecosystems, with many occupying an entire surveyed “quarter section” (one‐quarter of a square mile; 0.647 km^2^). Urbanized areas are few and small beyond the two towns of High River and Okotoks (total population approximately 36,000).

We selected 15 study sites adjacent to canola fields (“canola‐present”) and 15 sites adjacent to cereal fields (“canola‐absent”) at which to trap bees, using the road grid to position traps. Road allowances are public land in Alberta and facilitate access to many locations, enabling us to place traps along a gradient of canola cultivation intensity (which we define as the proportional cover of canola cultivation within a 250 m radius of the site). Canola‐present sites had 20%–93% canola cover (mean = 42%) within this radius, while canola‐absent sites had 0%–2% canola cover (mean = 0%). Sites were chosen to be at least 3 km apart (mean nearest neighbor distance = 4.3 km) by visiting a randomly ordered list of spaced locations on roads and selecting the first 30 locations that met the criteria. Although bumble bees have been located at distances over 10 km from their colony (Rao & Strange, 2012), most foraging activity occurs within 500 m of home (Osborne et al., 1999, 2008). Our site spacing reduces the likelihood that proximate traps capture foragers from the same central locations and helps ensure samples are independent.

The choice of sites was random across a region where nonagricultural land covers such as pastures and road margins are relatively even in their distribution (e.g., Figure 1). However, if either canola‐absent or canola‐present sites were, by chance, systematically closer to these seminatural covers, it is possible that bees may be influenced by the floral resources found in these cover types, rather than by the amount of canola. Alternatively, if either site type is systematically further from these land covers, bumble bees may forage at greater distances from their colony than they might otherwise, especially at times when floral resources are plentiful elsewhere. In both cases, this might lead to a bias in the trapping rate.

To test whether our site selection was unbiased with respect to nonagricultural land covers and therefore to the potential distribution of alternate floral resources, we conducted a landscape context analysis. We used land cover data representing all the major classes of seminatural features in the study area and found the proportional area of these features at multiple radii from each site (250, 500, 1,000 m, and where data were available 2,000 m). The area covered by (1) trees and shrubs, (2) wetlands, and (3) permanent or (4) recurring drainage features all of which may host floral resources either within or at their margins was manually digitized at the highest resolution available from Google Earth imagery up to a 1,000 m radius from each site (Google Earth, 2015, DigitalGlobe). Land cover products based on moderate resolution imagery (30 m; Agriculture and Agri‐Food Canada, 2015; Alberta Biodiversity Monitoring Institute, 2012) were used to obtain data for (5) road, rail, and linear feature margins which are typically vegetated and unmown for some or all of the season (S. Johnson, unpublished data); (6) grasslands and pastures which may retain floral resources when grazed at low intensities; (7) forage crops such as hay and alfalfa which may provide a temporary floral pulse; and, (8) urbanized sites which can include ornamental plantings and other vegetated microsites capable of attracting bees. We used a Mann–Whitney *U* test as a robust approach to compare the difference in proportional area between canola‐absent and canola‐present sites for each landscape variable.

### 2.2 Insect collection

Blue vane traps (SpringStar LLC, Woodinville, WA, USA) containing propylene glycol were placed at ground level in the road allowance close to the field edge, at least 2 m from the paved or graveled road surface. Traps were deployed continuously beginning on 23 June 2015 for 48 (±0.1 SE) days. In total, we collected 184 unique trapping events at 30 sites (several sites had additional traps added at their location beginning mid‐season), with a mean trapping event duration of 10 (±0.5 SE) days. Blue vane traps capture a broad spectrum of bee fauna, overlapping with pan trapping methods (Geroff, Gibbs, & McCravy, 2014) and can be untended for longer intervals when used with low evaporation preservation fluids. At each site, a trap was deployed continuously and emptied five times throughout the season to provide temporal resolution for bee phenology. The length of each trapping event (defined as the time interval between visits to empty a trap) was recorded to use as sampling effort covariate. Continuous sampling was intended to ensure that bumble bees, which are relatively infrequent visitors to insect traps, would be well sampled, and therefore that changes over time in worker and queen bumble bee abundances could be better captured. Our sampling activities in this region are unlikely to have impacts on population health, given the low spatial density of traps (>3 km spacing), and evidence that repeated sampling of a similar magnitude has no long‐term effect on pollinator populations (Gezon, Wyman, Ascher, Inouye, & Irwin, 2015).

In order to find an average timeline for canola floral availability in the study area, we recorded the proportion of canola plants in bloom at each visit to a canola‐present site by visually estimating bloom intensity on a 20‐point scale. We used a remote sensing‐based crop inventory for the same calendar year to determine the area of canola cover at a radius of 250 m from each trap (Agriculture and Agri‐Food Canada, 2015). The same measure at 500 m radius was highly correlated across all sites (Pearson's *r* = .96), but we opted for the more local measure to best characterize the immediate proximity of a trap to a canola field.

All bumble bee, genus *Bombus* (Hymenoptera: Apidae) specimens were identified to species. Overwintering bumble bee queens establish colonies earlier in the season with timing varying among species and locales (Williams, Thorp, Richardson, & Colla, 2014). In successfully established colonies, workers emerge next and provision the colony, with males and then new queens appearing at the end of the colony cycle. To distinguish among stages of colony development and demonstrate successful colony establishment, bumble bees were assigned to caste based on their length using published estimates of typical queen and worker sizes for each species (Williams et al., 2014). Male bumble bees were excluded due to their low sample size. We also identified to species and sex the sweat bee *Halictus rubicundus* (Hymenoptera: Halictidae)*,* the most abundant non‐*Bombus* social bee caught in our traps. Because this species is facultatively social, and therefore solitary in certain environments, we used diagnostic patterns in sex ratio throughout the flight season to verify its eusocial life history in our study area and therefore affirm its capacity to respond demographically within a season to a resource pulse (Soucy, 2002).

### 2.3 Abundance analyses

We analyzed changes in abundance throughout the season in relation to canola cultivation intensity using separate models for bumble bee queens, bumble bee workers, and *H. rubicundus*. All models had Julian day and canola cover as fixed effects. For bumble bees, we used generalized linear‐mixed models with random intercepts for species to reflect expected differences in abundance among species. Similarly, random Julian day slopes were chosen to model differences in the phenology of bumble bee species. We verified that a negative binomial error distribution in abundance was most appropriate for these data by comparing the fit of the relationship between mean and variance in species‐site abundance under the alternatives of Poisson's and quasi‐Poisson error distribution (Bolker et al., 2013). To control for differences among trapping events in sampling effort, abundance was modeled as a trapping rate, using the log of the deployment time as an offset term in the model. Julian day was included as a quadratic term, but in no case improved model fit, so was included only as a linear term. Due to the large number of zero abundance estimates, zero‐inflated models were tried first in all cases, and in none did they result in a significant reduction in deviance and were also omitted (Zuur, Ieno, Walker, Saveliev, & Smith, 2009). For all analyses, the full model with random intercepts and random slopes was compared using AIC to nested models with simpler random effects, and the best model was reported. Confidence intervals for model parameters were obtained using 400 bootstrap iterations. Julian day was scaled between 0 and 1, representing the start and end of sampling, respectively. An identical statistical methodology was used to further investigate the postflowering effects of canola on bee abundance. In this case, data were restricted to the last collection event of the season at the time of canola seed set (ranging from day 210 to 226 depending on the site; median collection date = 220).

### 2.4 Phenological analyses

We analyzed the relationship between the timing of bee collection and canola cultivation using several nonparametric approaches, running analyses separately for *H. rubicundus* and the queens and workers of the five most abundant bumble bee species. The dependent variable in these analyses was the Julian date of collection for each bee. To control for an uneven distribution of sampling effort over time, bee records were first rarefied by randomly removing bees of any species from a site in proportion to the number of hours of deployment during a sampling event, standardizing this to 6.8 days, which was the shortest sampling event duration. Two‐sample nonparametric tests were used to compare canola‐present and canola‐absent sites (shift of median collection date: Hodges–Lehmann, Mann–Whitney; change in collection date distribution: Kolmogorov–Smirnov). We used quantile regression to examine differences in the collection date distribution conditioning on canola cover. Quantile regression allows modeling of different quantiles of the dependent variable (Koenker, 2015), in this case, Julian day of collection. Here, we used it both because it is a robust regression method and to compare the relationship between Julian day and canola cover at quantiles representing the middle, early, and late stages of bee phenology (median, 25% and 75% quantiles, respectively).

All landscape and statistical analyses were conducted using base R v3.2.2 (R Core Team, 2016) packages with generalized linear‐mixed modeling using the lme4 package (Bates, Mächler, Bolker, & Walker, 2014) and quantile regression using the quantreg package (Koenker, 2015).

## 3 Results

We identified bees from 20 species in our traps (*N* = 1,978 bees). Six bumble bee species were singleton records and were removed, yielding data for 13 bumble bee (*Bombus* sp.) as well as *H. rubicundus* (Table S1). Solitary *H. rubicundus* produces a single brood that is slightly male biased, while in social populations, the first brood is female and the second, late season brood is predominantly male (Soucy, 2002). Fewer than 2% of the *H. rubicundus* bees we captured were male (*N* = 3) and these were in the second half of the flight season well after abundance had peaked (Table S1), suggesting populations in our study area are unlikely to be predominantly solitary.

Our continuous sampling spanned the start and end of bloom for the canola mass‐flowering crop. Field observations indicated that the peak bloom for canola fields in the study area was around day 190, 16 days after sampling began, with most fields having set seed around day 210, 16 days prior to the end of sampling activities (Fig. S1).

We verified the assumption that land covers potentially containing alternate floral resources were not systematically closer or further away from either canola‐present or canola‐absent sites (Table S2). Only one cover type did not meet this assumption (wetland cover within 250‐ and 500‐m scales). The small difference in median wetland cover between canola‐absent and canola‐present sites (3.7% at 250 m; 3.5% at 500 m) was largely attributable to one sampling location.

### 3.1 Temporal dynamics in abundance

**Figure 2:**
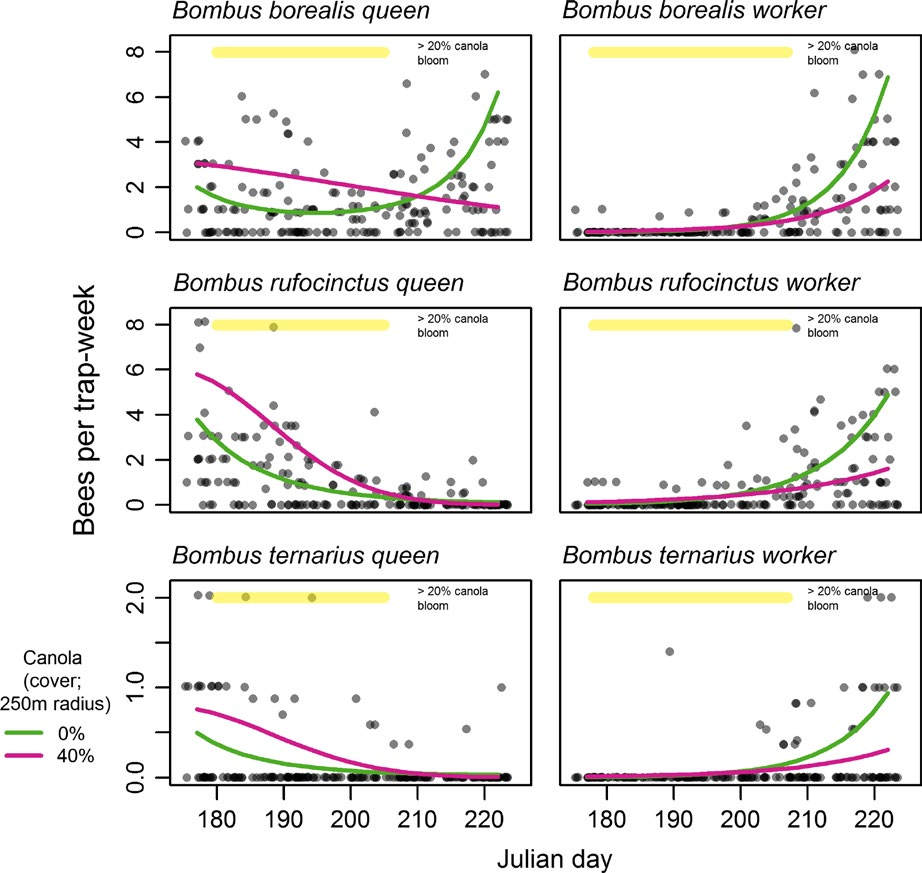
Within‐season changes in bumble bee abundance in relation to canola crops for three selected species using separate generalized linear‐mixed models for queens and workers. Interaction plots demonstrate a statistically significant interaction effect. 0% and 40% curves show the predicted interaction with canola cover at the average values for canola‐absent and canola‐present sites, respectively. Points indicate trapping rates at 184 trapping events and are shown as jittered semitransparent circles to reduce overplotting

**Figure 3:**
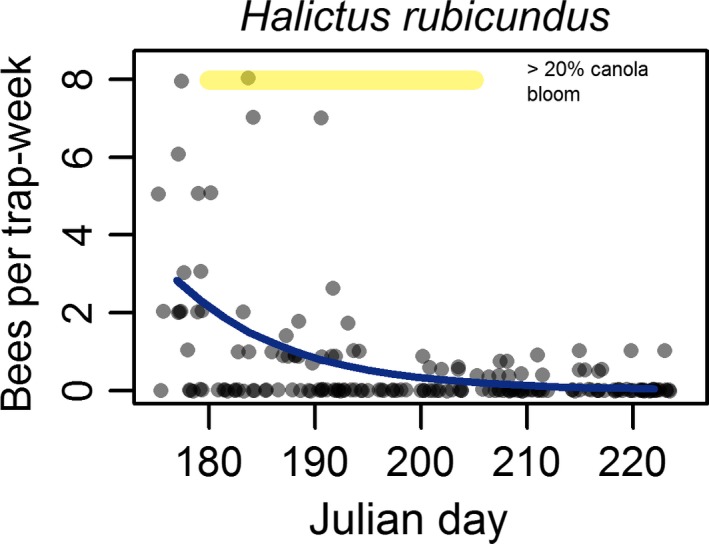
Within‐season changes in abundance for females of the facultatively social sweat bee, *Halictus rubicundus*. Fitted curve from a generalized linear model shows a statistically significant decrease in abundance over time at both canola‐present and canola‐absent sites. There was no interaction effect of canola cover. Points indicate trapping rates at 184 trapping events and are shown as jittered semitransparent circles to reduce overplotting

Worker bumble bees had reduced abundance where canola cover was higher. This effect was observed later in the season, reflecting an interaction between canola cover and Julian day (Figure 2; Table 1). In contrast to workers, queen bumble bees showed significantly higher abundance with higher canola cover, suggesting a positive response to the increased forage availability this represents (Figure 2; Table 1). However, there was an interaction driven by *B. borealis* where late‐season queens were less abundant in higher canola cover, mirroring the pattern seen in workers. The selected bumble bee models both included species random intercepts and slopes, and these had standard deviations significantly different from zero (Table 1), underlining the importance of accounting for interspecific differences in bumble bee phenology. The abundance of *H. rubicundus* was not affected by canola cover and showed a peak in abundance early in the season (Figure 3; Table S3).

**Table 1:** Within‐season changes in bumble bee abundance in relation to canola crops

	Queen bumble bees			Worker bumble bees		
	Value	*p*	Incidence rate ratio (bootstrap 95% CI)	Value	*p*	Incidence rate ratio (bootstrap 95% CI)
Intercept	**−9.759**	**<.001**		**−9.471**	**<.001**	
Canola cover (250 m)	**1.244**	**<.001**	**[1.341, 8.754]**	0.953	.132	[0.606, 7.882]
Julian day	−0.411	.728	[0.028, 11.09]	**2.581**	**<.001**	**[2.838, 58.90]**
Canola cover × Julian day	**−2.147**	**.003**	**[0.015, 0.578]**	**−2.150**	**.019**	**[0.019, 0.768]**
Species	13			13		
Trap events	184			184		
*N* (species × trap events)	2,392			2,392		
SD of abundance intercepts across species (bootstrap 95% CI)	**[1.444, 7.094]**			**[0.428, 2.118]**		
SD of Julian day slopes across species (bootstrap 95% CI)	**[0.306, 4.030]**			**[0.746, 3.332]**		

Estimated fixed effect parameters for generalized linear‐mixed modeling of abundance with a random intercept and Julian day slope for each species.

Bold indicates statistical significance at the alpha = 0.05 level.

After flowering had finished, higher canola cover was associated with a lower abundance of bumble bee workers. An increase from 0% to 100% in the proportion of canola cover within 250 m of a road allowance is associated with a 65% reduction in the trapping incidence rate of workers during the postflowering period (Table 2; β_1 _= −1.051). There was no difference in abundance observed in queens.

**Table 2:** Bumble bee abundance during the last sampling visit of the season, contrasting canola‐present fields where canola bloom had ended (<20% bloom) and canola‐absent fields

	Queen bumble bees			Worker bumble bees		
	Value	*p*	Incidence rate ratio (bootstrap 95% CI)	Value	*p*	Incidence rate ratio (bootstrap 95% CI)
Intercept	**−9.166**	**<.001**		**−7.130**	**<.001**	
Canola cover (250 m)	−1.152	.064	[0.069, 1.221]	**−1.051**	**.029**	**[0.119, 0.812]**
Species	13			13		
Trap events	47			44		
*N* (species × trap events)	611			572		
SD of abundance intercepts across species (bootstrap 95% CI)	**[0.961, 6.010]**			**[0.876, 2.531]**		
AIC	445.5			829.6		

Estimated fixed effect parameters for generalized linear‐mixed modeling of abundance with a random intercept for each species.

Bold indicates statistical significance at the alpha = 0.05 level.

To test whether this late‐season effect could be attributed to the small bias in wetland cover associated with canola‐absent sites, we repeated these analyses after removing data from the outlier wetland site and including wetland cover as a covariate. We again found a negative effect of canola cover, but no effect of wetland cover at 250‐m (Table S4) and 500‐m scales (results not presented).

### 3.2 Changes in phenology

Canola‐present sites had earlier median collection dates of workers in three bumble bee species and in queens of one (1–7 days earlier; Table S5a, b). Canola was also associated with differences in the collection date distribution in three of these cases (Table S5c), implying that canola presence (i.e., >20% canola cover within 250 m) was associated with more than a shift in the central tendency of collection dates, but also in the probability of collecting workers at different times throughout the season. One bumble bee species (*B. ternarius*) had a median worker collection date shift of 2 days later.

**Figure 4:**
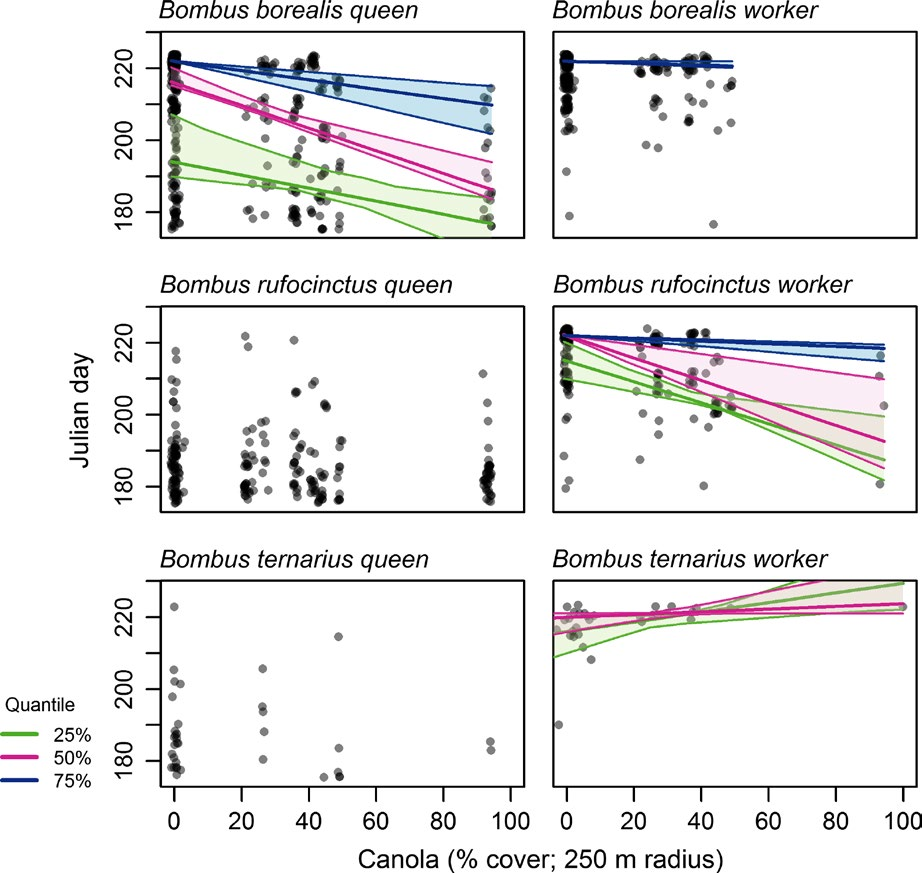
Relationship between canola cover and date of collection for three selected bumble bee species using quantile regression of rarefied data. Confidence intervals (95%; bootstrap) indicate the relationship for bees collected at the 25%, 50%, and 75% quantiles of the Julian day distribution, corresponding to the early, middle, and late periods in the phenology of each caste. Where there is no significant relationship, fits and confidence intervals have been omitted for clarity. Points indicate individual bee observations remaining after rarefaction to a constant sampling effort and are shown as jittered semitransparent circles to reduce overplotting

Using quantile regression to examine collection date distribution with canola as a continuous rather than categorical variable revealed that higher canola cover was most strongly associated with earlier collection dates of workers in *B. rufocinctus* and of queens in *B. borealis* (Figure 4; Table S6). Similar effects of canola were also recorded for the late‐season cohorts of *B. borealis* workers and *B. centralis* workers (Figure 4; Table S6; 75% quantile). The earliest cohort of *B. ternarius* was the only instance of a positive shift in worker collection date at higher canola cover (Figure 4; Table S6; 25% quantile). *H. rubicundus* had no evidence of a shift in median date when near canola fields (Table S5) nor of an association of its collection date distribution with canola cover (Fig. S2).

## 4 Discussion

Bumble bee workers were less abundant near canola (Tables 1 and 2; Figure 2) and had earlier median collection dates (Tables S5 and S6; Figure 4) at sites where this MFC was present (i.e., canola‐present sites; >20% canola cover within 250 m). The size of these effects increased under higher intensities of canola cultivation. Our finding of earlier collection dates for the three most abundant bumble bee species supports our hypothesis that food availability elicits a demographic response in colonies. It follows similar findings of earlier bumble bee observations in subalpine meadows with higher floral availability (Bowers, 1986). Others have also found responses in colony success near oilseed rape fields as measured by weight gain (Westphal et al., 2009). Our results are consistent with the hypothesis that MFCs support earlier colony establishment as measured by the earlier collection of workers at these sites (Tables S5 and S6; Figure 4), but there is no evidence that this may translate into higher colony or reproductive success. Rather, bumble bee species with earlier emergence near canola had reduced abundance at these locations later in the season (e.g., Table 2; Figure 2). We predicted that if food availability from canola produces a demographic response, sites with greater access to this resource should have more workers than sites where canola was scarce. Instead, we found that any colonies that may have been established near these places have fared worse, on average, than those established at greater distances from canola. However, in contrast to workers, early season queens were more abundant near canola (e.g., Table 2, Figure 2). This suggests a potential aggregative response, in that queens may be distributing themselves where forage is most available early in the season and that MFCs have the potential to influence where queens establish colonies.

As a stricter check of whether workers were affected by canola, we compared worker abundance at a time when canola bloom had largely finished in this agroecosystem. This check made the assumption that canola‐absent and canola‐present sites have similar distributions of wildflower availability in their vicinity, and therefore that after canola flowering has ended, workers are less likely to forage far from their colony. This was performed to maximize the likelihood that workers captured at a site came from a colony near that site. We again found that there were fewer workers near canola in this restricted test (Tables 2 and S4).

Our verification of the consistency of landscape conditions across study sites (Table S2) increases our confidence that this effect of reduced worker abundance and earlier collection dates is associated with canola cover, rather than to other floral resources or to unmeasured variables, such as the availability of nesting sites. We have shown that there is variation in these unmeasured variables, but this variation is consistent across all 30 sites, which effectively controls for its influence. The negative effect of canola cover on bumble bee workers after canola bloom is therefore unlikely to reflect workers foraging further afield, given that we expect all sites to have an unbiased distribution of near and far floral resources.

We elected to examine *H. rubicundus*, a social bee in our study area, because, like bumble bees, this colonial species has the potential to respond demographically to a midseason pulse in floral availability. It was not collected earlier at canola fields nor did it increase in abundance, as predicted, with canola cropping intensity. In contrast to bumble bees, there was no evidence that this species was affected positively or negatively by canola cover at any stage of colony development. This may reflect the critical colony growth phase occurring before bloom had begun, suggested in our results by highest abundance early in the season (Figure 3). Additionally, these are smaller‐bodied bees and consequently tend to forage closer to their nests (Zurbuchen et al., 2010). Although this species is polylectic and there is evidence that these bees visits plants in Brassicaceae (Brittain & Newton, 1933), in which canola is a member, they may be less able than bumble bees to move to the location of a resource pulse that varies spatially and unpredictably from season to season.

Seminatural and uncultivated areas that provide alternative forage sources are infrequent in this intensively managed agroecosystem. The grid of minor roads and tracks that covers much of the Canadian Prairies is therefore an important remnant habitat, with the unmown seminatural margins of these abundant features providing a consistent source of forage throughout the season (S. Johnson, unpublished data). Due to the much larger land surface in this study area covered by canola than by road margins, this MFC is likely to be a dominant source of flowers when the crop is in bloom. This raises the possibility that higher MFC densities cause crowding of bumble bees near the spatially limited road allowances, resulting in less successful colonies and lower abundance near these crops when the bloom ends. A scenario where a greater area of MFC attracts a higher density of queens seeking to establish colonies, but colonies are less successful, is consistent with the pattern we found. For example, in the most abundant bumble bee species (*B. borealis*), queens were more abundant at canola‐present sites throughout the season, but late season workers were fewer. If a higher than usual proportion of these queens established colonies because of canola food availability, crowding of these colonies, and therefore competition for late‐season resources after canola bloom has ended, may explain the deficit in workers we found at these sites. The importance of late‐season flowers for bumble bees in agroecosystems dominated by oilseed rape has been previously demonstrated (Westphal et al., 2009) and may in our system also be a limiting resource. Further investigations, for example, using experimental colonies, are required to conclusively demonstrate competition as a mechanism influencing colony success in this system.

In summary, our results demonstrate that MFCs such as canola when grown at high cropping intensities are related to an earlier demographic response and a lower late‐season abundance of bumble bee workers and that this latter effect is not due to the redistribution of bees to more abundant resources. Our results also suggest that minimizing the localized deficits of bumble bees created by canola could be achieved through a spatial optimization of field types. The area of canola planted in the vicinity of our traps was related to the magnitude of the collection date shift and the reduction in worker abundance. Any negative impacts of an MFC may therefore be reduced by spacing fields more evenly across the agricultural landscape, reducing aggregation of the large and transient pollinator resource. Conservation measures that should be evaluated experimentally include decreasing field size, avoiding planting MFCs in two adjacent fields, and encouraging unmown strips and floral plantings on road allowances to both dilute the intensive cover of canola and boost late‐season floral resources.

In contrast to European studies of oilseed rape, the conservation implications of North American canola crops for pollinators has received relatively little attention (but see Morandin & Winston, 2005, 2006; Morandin, Winston, Abbott, & Franklin, 2007). Yet, canola is de facto one of North America's major land covers, with approximately 85,000 km^2^ planted in this crop in 2015 alone, and tens of thousands of additional square kilometers planted in annual rotation. The consistency of agricultural practices across this vast region means that small changes to the pattern and spatial intensity of canola cultivation, such as those we have proposed, could have widespread conservation benefits for North American bumble bee populations.

## Conflict of interest

None declared.

## Data sources

All specimens used in this study have been deposited in the insect collection at the University of Calgary.
