# Supplementary material for: Reduced abundance and earlier collection of bumble bee workers under intensive cultivation of a mass‐flowering prairie crop
Source: Ecol Evol. 2017 Mar 12;7(7):2414–22. doi: 10.1002/ece3.2856 (PMC5383479; doi:10.1002/ece3.2856)
Supplement: Supplementary file 1 [file ECE3-7-2414-s001.docx]

Figure S1. Estimated proportion of plants in a canola field that are in bloom by Julian day. Points are fields adjacent to sampling sites during each trap-event (N=184), and are shown as jittered semi-transparent circles to reduce over-plotting. LOESS regression fit (green curve) estimates mean canola flowering phenology in the study area during 2015.

**
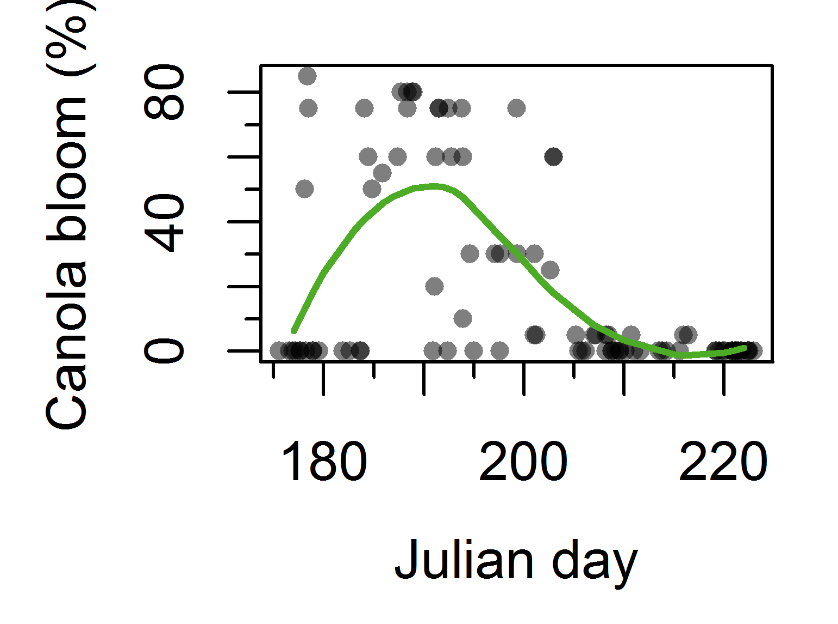
**

Figure S2. No significant relationship between canola cover and collection date for the facultatively social sweat bee, *H. rubicundus*, analyzed using quantile regression of rarefied data. Quantile regression conducted at 25%, 50%, and 75% quantiles of the Julian day distribution, corresponded to the early, middle and late periods in the phenology of each species. Points indicate individual bee observations remaining after rarefaction to a constant sampling effort and are shown as jittered semi-transparent circles to reduce over-plotting.


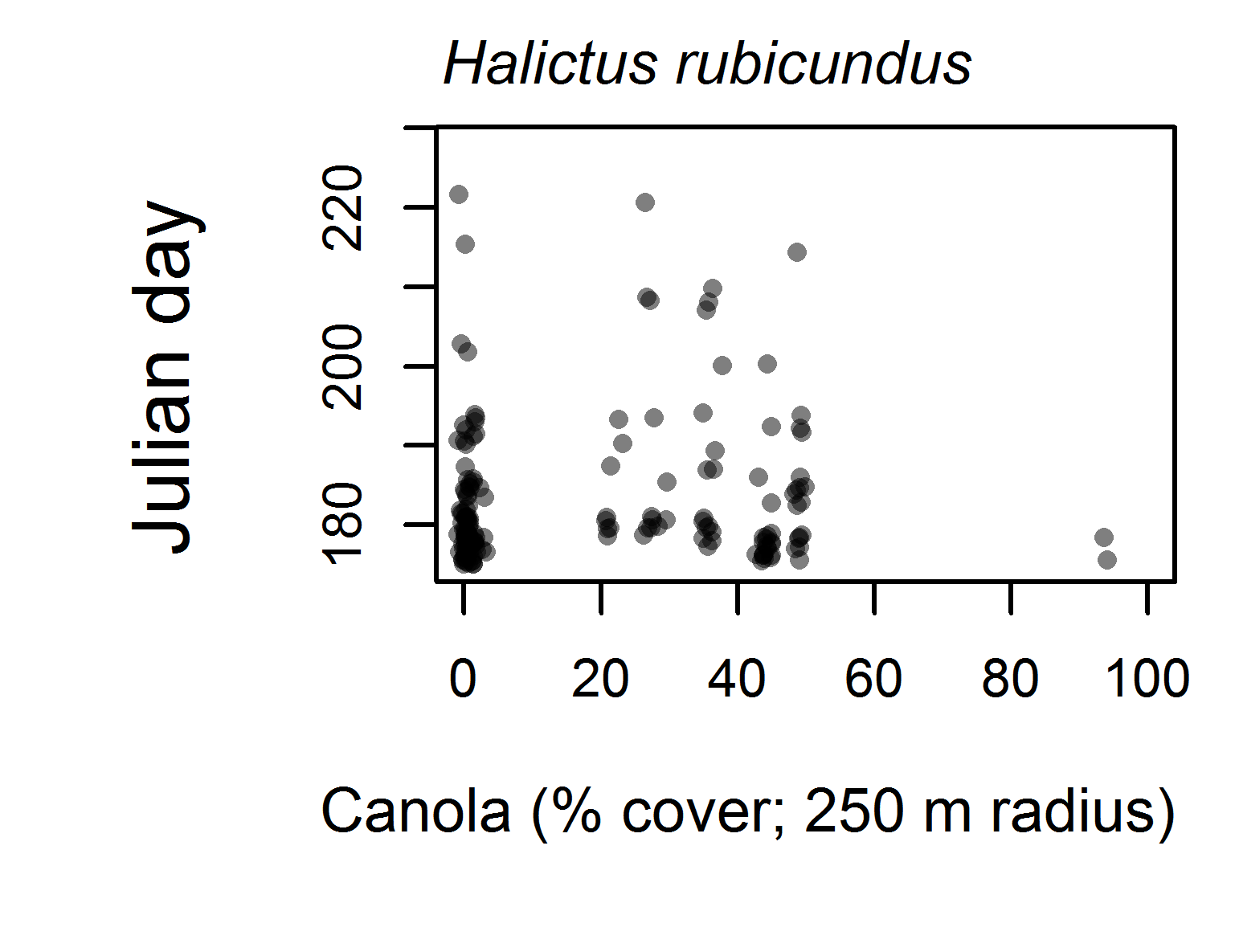


Table S1. Bees sampled by species and trapping event (where an event is one of a sequence of collections from a continuously-deployed trap), ordered from most to least abundant species.

|  |  |  |  | Bees trapped | | | | |
| --- | --- | --- | --- | --- | --- | --- | --- | --- |
|  | Total bees trapped |  |  | Event 1 | Event 2 | Event 3 | Event 4 | Event 5 |
| **Bumble bees**  *Bombus borealis* | 870 |  | queen | 56 | 49 | 34 | 171 | 202 |
|  |  |  | worker | 2 | 1 | 3 | 91 | 261 |
| *Bombus rufocinctus* | 564 |  | queen | 105 | 91 | 21 | 35 | 8 |
|  |  |  | worker | 4 | 2 | 6 | 144 | 148 |
| *Bombus centralis* | 120 |  | queen | 4 | 0 | 1 | 4 | 2 |
|  |  |  | worker | 3 | 14 | 14 | 53 | 25 |
| *Bombus ternarius* | 89 |  | queen | 14 | 12 | 4 | 4 | 2 |
|  |  |  | worker | 0 | 2 | 0 | 12 | 39 |
| *Bombus nevadensis* | 49 |  | queen | 6 | 5 | 1 | 12 | 10 |
|  |  |  | worker | 1 | 2 | 0 | 6 | 6 |
| *Bombus occidentalis* | 27 |  | queen | 0 | 1 | 0 | 1 | 1 |
|  |  |  | worker | 0 | 0 | 0 | 8 | 16 |
| *Bombus fervidus* | 21 |  | queen | 0 | 4 | 1 | 4 | 1 |
|  |  |  | worker | 0 | 1 | 2 | 6 | 2 |
| *Bombus mixtus* | 19 |  | queen | 0 | 0 | 0 | 0 | 0 |
|  |  |  | worker | 0 | 1 | 2 | 13 | 3 |
| *Bombus vagans* | 9 |  | queen | 0 | 0 | 0 | 0 | 0 |
|  |  |  | worker | 3 | 1 | 1 | 1 | 3 |
| *Bombus flavifrons* | 8 |  | queen | 0 | 0 | 0 | 0 | 0 |
|  |  |  | worker | 0 | 0 | 1 | 3 | 4 |
| *Bombus frigidus* | 5 |  | queen | 0 | 0 | 0 | 0 | 0 |
|  |  |  | worker | 1 | 1 | 0 | 0 | 3 |
| *Bombus huntii* | 5 |  | queen | 0 | 0 | 0 | 0 | 0 |
|  |  |  | worker | 1 | 0 | 0 | 2 | 2 |
| *Bombus insularis* | 2 |  | queen | 0 | 1 | 0 | 0 | 1 |
|  |  |  | worker | 0 | 0 | 0 | 0 | 0 |
|  |  |  |  |  |  |  |  |  |
| **Other social bees**  *Halictus rubicundus* | 190 |  | female | 115 | 30 | 18 | 16 | 8 |
|  |  |  | male | 0 | 0 | 0 | 2 | 1 |
|  |  |  |  |  |  |  |  |  |
| Totals: | 1978 |  |  | 315 | 218 | 109 | 588 | 748 |

Table S2. Landscape context analyses, testing for median differences in land cover potentially containing late-season floral resources surrounding sampling sites, using a Mann-Whitney test. Canola-absent sites have a higher area of wetlands at two scales, but otherwise show no significant difference from canola-present sites in terms of potential forage availability.

|  | Median percent area of land cover  within a circle of given radius | | | |  | | | |
| --- | --- | --- | --- | --- | --- | --- | --- | --- |
| Radius from site |  | Canola-present sites (N=15) | | Canola-absent  sites (N=15) | |  | Mann-Whitney P | |
| **Drainage features (permanent) and adjacent riparian corridor ^1^** | | | | | | | |  |
| < 2000 m | 0.0 | | 0.0 | |  | | |  |
| **Drainage features (recurring) and coulees ^1^** | | | | | | | |  |
| < 1000 m | 0.0 | | 0.0 | |  | | |  |
| 2000 m | 2.4 | | 1.7 | | 0.836 | | |  |
| **Road, rail, and linear feature margins (typically vegetated) ^2^** | | | | | | | |  |
| 250 m | 19.2 | | 18.3 | | 0.917 | | |  |
| 500 m | 10.5 | | 11.9 | | 0.329 | | |  |
| 1000 m | 6.9 | | 9.7 | | 0.093 | | |  |
| 2000 m | 6.0 | | 7.0 | | 0.384 | | |  |
| **Grasslands (including pastures) ^3^** | | | | | | | |  |
| 250 m | 0.5 | | 19.7 | | 0.060 | | |  |
| 500 m | 10.3 | | 20.2 | | 0.219 | | |  |
| 1000 m | 14.8 | | 25.9 | | 0.507 | | |  |
| 2000 m | 22.8 | | 15.8 | | 0.590 | | |  |
| **Forage crops (e.g. hay, alfalfa) ^3^** | | | | | | | |  |
| < 500 m | 0.0 | | 0.0 | |  | | |  |
| 1000 m | 0.6 | | 1.9 | | 0.834 | | |  |
| 2000 m | 4.0 | | 2.9 | | 0.787 | | |  |
| **Urbanized (including agricultural, residential and industrial sites) ^2^** | | | | | | | |  |
| 250 m | 0.0 | | 0.0 | |  | | |  |
| 500 m | 0.2 | | 0.0 | | 0.642 | | |  |
| 1000 m | 0.7 | | 1.1 | | 0.883 | | |  |
| 2000 m | 0.9 | | 1.0 | | 0.803 | | |  |
| **Trees and shrubs  ^1^** | | | | | | | |  |
| < 500 m | 0.0 | | 0.0 | |  | | |  |
| 1000 m | 0.3 | | 0.3 | | 0.867 | | |  |
| **Wetlands (permanent or recurring) ^1^** | | | | | | | |  |
| 250 m | 0.0 | | 3.7 | | **0.015** | | |  |
| 500 m | 0.2 | | 3.7 | | **0.036** | | |  |
| 1000 m | 1.9 | | 3.5 | | 0.383 | | |  |

**^1^**Data source: prepared by manual object identification within 1000 m of each site using Google Earth imagery at the highest resolution available (Google, 2015. DigitalGlobe)

**^2^**Data source: Alberta Human Footprint layer (ABMI, 2012). Based on 30 m imagery, in part.

**^3^**Data source: Crop Inventory (Agricultural and Agri-Food Canada, 2015). Based on 30 m imagery.

Table S3. Within-season changes in abundance for the facultatively social sweat bee *H. rubicundus* in relation to canola crops. Estimated parameters from a generalized linear model.

|  | *Halictus rubicundus* | | |  |
| --- | --- | --- | --- | --- |
|  | Value | P | Incidence Rate Ratio (Profile 95% CI) |  |
| Intercept | **-3.759** | **< 0.001** |  |  |
| Canola cover (250 m) | -0.785 | 0.375 | [0.081, 2.806] |  |
| Julian day | **-4.692** | **< 0.001** | **[0.002, 0.031]** |  |
| Canola cover x Julian day | 1.184 | 0.579 | [0.040, 198.269] |  |
|  |  |  |  |  |
| N | 184 |  |  |  |

Bold indicates statistical significance at the alpha=0.05 level

Table S4. Bumble bee abundance during the last sampling visit of the season, contrasting canola-present fields where canola bloom had ended (< 20% bloom) and canola-absent fields including a potentially confounding effect of wetland cover (compare with Table 2). There is no evidence for an influence of wetlands, with the effect of canola cover remaining significant for workers. Overall these models are poorer fits than those excluding the wetland cover variable, with larger confidence intervals and higher AICs. Estimated fixed effect parameters for generalized linear mixed modelling of abundance with a random intercept for each species.

|  | Queen bumble bees | | |  | Worker bumble bees | | |
| --- | --- | --- | --- | --- | --- | --- | --- |
|  | Value | P | Incidence Rate Ratio (Bootstrap 95% CI) |  | Value | P | Incidence Rate Ratio (Bootstrap 95% CI) |
| Intercept | **-9.292** | **< 0.001** |  |  | **-7.133** | **< 0.001** |  |
| Canola cover (250m) | -0.878 | 0.195 | [0.058, 1.901] |  | **-1.046** | **0.050** | **[0.117, 0.895]** |
| Wetland cover (250m) | 0.932 | 0.348 | [0.056, 20.637] |  | 0.031 | 0.983 | [0.061, 7.118] |
|  |  |  |  |  |  |  |  |
| Species | 13 |  |  |  | 13 |  |  |
| Trap-events | 47 |  |  |  | 44 |  |  |
| N (Species x Trap-events) | 611 |  |  |  | 572 |  |  |
| SD of abundance intercepts across species  (Bootstrap 95% CI) | **[2.344, 430.010]** | |  |  | **[2.552, 14.492]** | |  |
| AIC | 446.7 | |  |  | 831.6 | |  |
|  |  | |  |  |  | |  |

Bold indicates statistical significance at the alpha=0.05 level

Table S5. Two-sample non-parametric tests of difference in Julian day distribution of bees collected, comparing canola-present and canola-absent fields. Tests of location shift (a and b) and difference in distribution (c) indicate significantly earlier median dates for *B. borealis* and *B. rufocinctus*, and a later median date for *B. ternarius* collected near canola fields. Data were rarefied to constant sampling effort.

|  |  |  | (a)Hodges-Lehmann  median shift | |  | (b)Mann-Whitney  median shift | |  | (c)Kolmogorov-Smirnov distribution difference | |
| --- | --- | --- | --- | --- | --- | --- | --- | --- | --- | --- |
|  |  |  | (days) | 95% CI |  | W | P |  | D | P |
| **Bumble bees**  *Bombus borealis* | queen |  | **-7** | **[-12, -3]** |  | **11053.5** | **< 0.001** |  | **0.304** | **< 0.001** |
|  | worker |  | **-1** | **[-1, 0]** |  | **5763.5** | **0.011** |  | **0.361** | **< 0.001** |
| *Bombus rufocinctus* | queen |  | 0 | [-3, 0] |  | 5703 | 0.365 |  | 0.106 | 0.583 |
|  | worker |  | **-2** | **[-8, -1]** |  | **2172.5** | **< 0.001** |  | **0.561** | **< 0.001** |
| *Bombus centralis* | queen |  | 12 | [-38, 42] |  | 7 | 0.857 |  | 0.417 | 0.886 |
|  | worker |  | **-6** | **[-12, 0]** |  | **404** | **0.031** |  | 0.318 | 0.067 |
| *Bombus ternarius* | queen |  | 0 | [-7, 7] |  | 141.5 | 0.803 |  | 0.094 | 1.000 |
|  | worker |  | **2** | **[0, 5]** |  | **201.5** | **0.048** |  | **0.542** | **0.018** |
| *Bombus nevadensis* | queen |  | 0 | [-13, 18] |  | 86.5 | 0.845 |  | 0.231 | 0.866 |
|  | worker |  | 0 | [-23, 20] |  | 17 | 0.931 |  | 0.25 | 0.996 |
| **Other social bees**  *Halictus rubicundus* |  |  | 0 | [0, 2] |  | 4212.5 | 0.095 |  | 0.156 | 0.254 |
|  |  |  |  |  |  |  |  |  |  |  |

Bold indicates statistical significance at the alpha=0.05 level

Table S6. Results of quantile regressions on Julian day for five most abundant bumble bees included in this study, and the sweat bee *H. rubicundus*. Data were rarefied to constant sampling effort.

|  |  |  | Julian day |  | Canola cover (250 m) | |
| --- | --- | --- | --- | --- | --- | --- |
|  |  | Quantile | Intercept |  | Coefficient | P |
| **Bumble bees**  *Bombus borealis* | queen | 25% | 194 |  | **-18.181** | **0.007** |
|  |  | 50% | 216 |  | **-31.488** | **< 0.001** |
|  |  | 75% | 222 |  | **-12.960** | **< 0.001** |
|  | worker | 25% | 216 |  | -2.039 | 0.819 |
|  |  | 50% | 222 |  | -5.594 | 0.077 |
|  |  | 75% | 222 |  | **-2.693** | **0.001** |
| *Bombus rufocinctus* | queen | 25% | 179 |  | 0.000 | 1.000 |
|  |  | 50% | 184 |  | 0.000 | 1.000 |
|  |  | 75% | 189 |  | -5.347 | 0.022 |
|  | worker | 25% | 215 |  | **-29.239** | **< 0.001** |
|  |  | 50% | 222 |  | **-31.167** | **< 0.001** |
|  |  | 75% | 222 |  | **-3.761** | **0.009** |
| *Bombus centralis* | queen | 25% | 179 |  | -4.498 | 0.939 |
|  |  | 50% | 180 |  | 51.175 | 0.509 |
|  |  | 75% | 215 |  | 17.044 | 0.840 |
|  | worker | 25% | 198 |  | -22.458 | 0.183 |
|  |  | 50% | 210 |  | -11.093 | 0.399 |
|  |  | 75% | 216 |  | **-30.680** | **0.007** |
| *Bombus ternarius* | queen | 25% | 179 |  | -2.058 | 0.701 |
|  |  | 50% | 184 |  | 0.000 | 1.000 |
|  |  | 75% | 198 |  | -15.046 | 0.462 |
|  | worker | 25% | 216 |  | **13.467** | **0.017** |
|  |  | 50% | 220 |  | 3.761 | 0.419 |
|  |  | 75% | 222 |  | 0.000 | 1.000 |
| *Bombus nevadensis* | queen | 25% | 184 |  | 4.116 | 0.837 |
|  |  | 50% | 207 |  | -5.347 | 0.790 |
|  |  | 75% | 221 |  | -6.948 | 0.599 |
|  | worker | 25% | 191 |  | 24.698 | 0.563 |
|  |  | 50% | 216 |  | -26.756 | 0.492 |
|  |  | 75% | 216 |  | 14.544 | 0.727 |
| **Other social bees**  *Halictus rubicundus* |  | 25% | —^a^ |  | —^a^ | —^a^ |
|  |  | 50% | 179 |  | 0.000 | 1.000 |
|  |  | 75% | 184 |  | 8.612 | 0.291 |

Bold indicates where the bootstrapped 95% confidence interval of the coefficient does not include zero.

^a^Indicates failure of quantile regression fit
